# Indication criteria for total hip or knee arthroplasty in osteoarthritis: a state-of-the-science overview

**DOI:** 10.1186/s12891-016-1325-z

**Published:** 2016-11-09

**Authors:** Maaike G. J. Gademan, Stefanie N. Hofstede, Thea P. M. Vliet Vlieland, Rob G. H. H. Nelissen, Perla J. Marang-van de Mheen

**Affiliations:** 1 Department of Orthopaedics, Leiden University Medical Center, Albinusdreef 2, Leiden, 2333 ZA The Netherlands; 2 Department of Clinical Epidemiology, Leiden University Medical Center, Albinusdreef 2, Leiden, 2333 ZA The Netherlands; 3 Department of Medical Decision Making, Leiden University Medical Center, Albinusdreef 2, Leiden, 2333 ZA The Netherlands; 4 Department of Orthopaedics, P.O. Box 9600, 2300 RC Leiden, The Netherlands; 5 Department of Clinical Epidemiology, P.O. Box 9600, 2300 RC Leiden, The Netherlands; 6 Rijnlands Rehabilitation Center, Leiden, The Netherlands; 7 Sophia Rehabilitation, The Hague, The Netherlands

## Abstract

**Background:**

This systematic review gives an overview of guidelines and original publications as well as the evidence on which the currently proposed indication criteria are based. Until now such a state-of-the-science overview was lacking.

**Methods:**

Websites of orthopaedic and arthritis organizations (English/Dutch language) were independently searched by two authors for THA/TKA guidelines for OA. Furthermore, a systematic search strategy in several databases through August 2014 was performed. Quality of the guidelines was assessed with the AGREE II instrument, which consists of 6 domains (maximum summed score of 6 indicating high quality). Also, the level of evidence of all included studies was assessed.

**Results:**

We found 6 guidelines and 18 papers, out of 3065 references. The quality of the guidelines summed across 6 domains ranged from 0.46 to 4.78. In total, 12 THA, 10 TKA and 2 THA/TKA indication sets were found. Four studies stated that no evidence-based indication criteria are available. Indication criteria concerning THA/TKA consisted of the following domains: pain (in respectively 11 and 10 sets), function (12 and 7 sets), radiological changes (10 and 9 sets), failed conservative therapy (8 and 4 sets) and other indications (6 and 7 sets). Specific cut-off values or ranges were often not stated and the level of evidence was low.

**Conclusion:**

The indication criteria for THA/TKA are based on limited evidence. Empirical research is needed, especially regarding domain specific cut-off values or ranges at which the best postoperative outcomes are achieved for patients, taking into account the limited lifespan of a prosthesis.

**Electronic supplementary material:**

The online version of this article (doi:10.1186/s12891-016-1325-z) contains supplementary material, which is available to authorized users.

## Background

Total hip and knee arthroplasty (THA/TKA) have been widely performed since the 1970s. In 2009 over a million of THA and TKA were carried out in the United States [1]. Osteoarthritis (OA) is the main clinical indication for which these procedures are performed [2]. Due to the ageing society as well as the obesity epidemic, the prevalence of OA is increasing [3]. As a result the procedure rates of THA and TKA are expected to rise, some estimates even indicate a quadruple demand by 2030 [4, 5].

The rise in THA/TKA surgery has important implications for health care costs as well as capacity. As such, it is of utmost importance that patients are carefully selected, and that these procedures are optimally timed to achieve the best possible patient outcomes and that revision surgery is prevented thereby reducing costs and worse outcomes. However, large heterogeneity exists in the patients disease severity at the time of surgery [6, 7]. This can partly be explained by the patient’s own wishes, with some patients preferring a THA/TKA to continue an active lifestyle whereas others request surgery to be able to perform daily living activities. In addition, the attitude of the surgeon towards arthroplasty plays an important role since surgeons with a positive attitude towards this procedure will have higher surgery rates. This may explain why studies on appropriateness criteria for THA/TKA showed that in approximately 20-45 % of patients appropriateness of arthroplasty was considered uncertain [8–11]. All of these may explain to some extent why a substantial proportion of the patients is unsatisfied after THA and TKA (10-30 %), indicating that outcomes are less than expected and/or that expectations were too high [12]. Therefore, evidence-based indication criteria for THA/TKA to guide decision making are warranted to improve optimal timing and patient selection, which is internationally acknowledged [13–18].

Guidelines concerning THA and TKA indications have been published and several studies regarding the appropriateness of THA and TKA have been conducted [3, 13, 19–21]. However, an overview of the evidence on which the proposed indication criteria are based is lacking, to guide decision making on timing of THA and TKA. In the present study the available guidelines and their indication sets for primary THA and TKA will be reviewed. In addition, we assess the quality of these guidelines and the evidence on which the indication sets are based. In the second part a systematic search is conducted of scientific publications containing proposed indication sets for primary THA and TKA in OA or expert opinion.

## Methods

### Search strategy

Websites of orthopaedic and arthritis organizations (English or Dutch websites) were independently searched by two authors for guidelines concerning primary THA/TKA for OA. When these websites cross-linked to guidelines from other organizations these were also included. All available guidelines published since January 1, 2000 were included. A librarian-assisted search strategy was performed on August 3 2014 to retrieve additional publications on THA/TKA indications. The following databases were searched: Pubmed, MEDLINE, Embase, Web of Science, the COCHRANE Librabry, CENTRAL and CINAHL. Searches were limited to English, Dutch and German language papers published since January 1, 2000 (see Additional file 1).

### Selection of publications

First titles and abstracts were independently screened by two authors (MG/SH).The full-text articles were reviewed by MG and were included when the following criteria were met: studies reporting about indication criteria and/or appropriateness of decision tools for primary THA/TKA in OA. Papers involving guidelines on unicompartimental replacements, resurfacing or revision of THA/TKA were excluded if no separate indications for primary THA/TKA were provided. Also papers on prioritizing tools to reduce waiting times were excluded unless these reported on the appropriateness of surgery as all of these patients on the waiting list already have an indication and variables determining priority are not necessarily the same as variables determining an indication for surgery.

The included papers where checked by a second author (SH). If disagreement existed the authors tried to reach consensus, when necessary a third author had the decisive vote (PM). When a guideline was also published as a scientific paper, only the guideline was included.

### Data extraction

The following information was extracted from the guidelines by MG: orthopaedic or arthritis organization, publication date, indication criteria and the level of evidence on which indication criteria were based (see below). We extracted the following information from the publications: first author, publication date, country where the indication criteria were developed, the organization(s) that initiated the development of the criteria, study type, indication criteria and the level of evidence on which indication criteria were based. The level of evidence was scored according to the following the criteria [3]:

- ➢ Ia evidence from meta-analysis of randomized controlled trials
- ➢ Ib evidence from at least one randomized controlled trial
- ➢ IIa evidence from at least one controlled study without randomization
- ➢ IIb evidence from at least one well-designed quasi-experimental study
- ➢ III evidence from at least one non-experimental descriptive study, such as comparative studies, correlation studies, and case-control studies
- ➢ IV evidence from expert committee reports or opinions or clinical experience of respected authorities or both

Data extraction and level of evidence score was checked by SH.

### Quality of the guidelines

Guideline quality was assessed with the validated AGREE-II instrument (Appraisal of Guidelines for Research and Evaluation, Dutch version) [22]. This instrument evaluates the process of practice guideline development and the quality of reporting. Two authors independently scored the guidelines according to the AGREE-II protocol (MG/SH). When large differences existed the authors tried to reach consensus, when necessary a third author had the decisive vote (PM).

The AGREE-II consists of six quality domains: 1) scope and purpose, 2) stakeholder involvement, 3) rigour of development, 4) clarity of presentation, 5) applicability and 6) editorial independence. Each domain entails several questions which are rated from 1 (lowest score) to 7 (highest score), with 1 rated for items with no clear discussion or no specific information, 7 for exceptional reporting quality, 2–6 for items not fully meeting the AGREE-II criteria. Scaled domain scores were calculated using the following formula:

$$ \frac{\left(\mathrm{Obtained}\ \mathrm{score}\ \hbox{-}\ \mathrm{Minimum}\ \mathrm{possible}\ \mathrm{score}\right)}{\left(\mathrm{Maximum}\ \mathrm{possible}\ \mathrm{score}\ \hbox{-}\ \mathrm{Minimum}\ \mathrm{possible}\ \mathrm{score}\right)} $$

The scores will always lie between 0 and 1, with scores closer to 1 indicating higher quality. The scaled domain scores from the two authors were averaged to obtain one quality score for each domain. We summed the scaled domain scores across the 6 domains to obtain 1 overall guideline score. The maximum summed score was thus 6, indicating high quality.

## Results

Across guidelines and studies, 12 THA, 10 TKA and 2 THA/TKA indication sets were found.

### Guidelines

We found six guidelines concerning THA, of which three specific OA guidelines (EULAR [20], NICE [23] and OARSI [3]). In addition, five guidelines concerning TKA were found, of which four OA specific guidelines (BOA [24], EULAR [19], NICE [23] and OARSI [3]) (Table 1).

**Table 1:** Guidelines and their indication criteria concerning total hip arthroplasty and total knee arthroplasty

Guideline	Year of publication	OA specific	Evidence	Indication criteria				
				Pain	Function	Radiological changes	Failed or futile conservative therapy	Other criteria
Knee								
British Orthopedic Association [24]	2013	Yes	Level IV	Moderate or severe pain		KL > III in at least one of the knee joints compartments	Yes	Patients outside these criteria may still be considered for surgery but a second opinion/ recorded case discussion is advised. Cases focus on patients without pain (primary indication) but who present with: functional disability in the presence of end stage cartilage disease. Progressive deformity of the knee (varus/valgus) with functional disability.
Eular [19]	2003	Yes	Level IV	Refractory pain	Disability	Radiological evidence of knee OA		
NZ [40]	?	No, but based on BOA guidelines which is OA specific	Level IV	Severe pain	Disability	Radiological changes	Yes	Occasionally there may be an indication to replace a knee because of progressive deformity and/or instability, and pain may not necessarily be the most significant factor. Where comorbidities exist risk benefit considerations may rule out the operation in an individual patient.
Hip								
British Orthopedic Association [41]	2013	No	Level IV	Inadequately controlled by medication	Restriction in function	Narrowing of the joint space	Yes	Compromised quality of life
Eular [20]	2005	Yes	Level IV	Refractory pain	Disability	Radiological evidence of hip OA		
NOV [42]	2010	No	Level IV	Pain	Function loss	Radiological changes	Yes	Younger age and obesity are relative contraindications. Delay of surgery in high age is not advisable in view of reduced functional outcome and increased mortality. In addition when progressive loss of function (with or without contractures) predominates over pain, surgery should not be delayed in view of reduced postoperative functional outcome.
NZ [43]	?	No	Level IV	Significant pain	Disability	Radiological changes	Yes	
Joint								
OARSI (hip-knee) [3]	2008	Yes	Level IV	No adequate pain relief	No adequate functional improvement		Yes	
NICE [23]	2014	Yes	Level IV	Pain	Stiffness and reduced function		Yes	Substantial impact on quality of life

Legend to Table 2: *KL* Kellgren Lawrence, *OA* osteoarthritis

#### Indication criteria concerning THA and TKA

Most indication criteria consisted of the following three domains: pain, function and radiological changes, with the prerequisite that pain could not be controlled by conservative therapy (Table 1). Specific cut-off values or ranges for pain and function were not reported. For radiological changes only the BOA TKA guideline reported a cut-off value (Kellgren Lawrence grade ≥ III). The evidence on which the indication criteria were based was rated as low quality evidence (level IV).

#### Quality of the guidelines

**Fig. 1:**
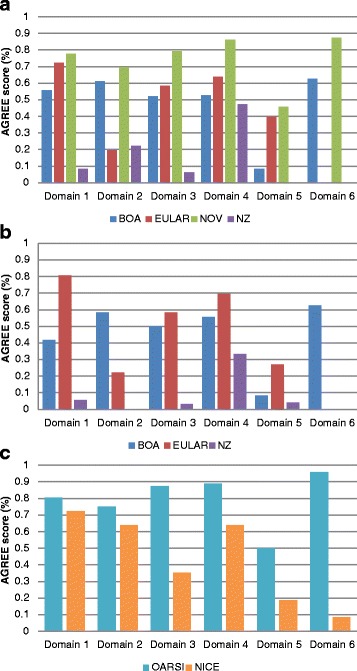
AGREE II guideline quality scores. Panel (**a**) AGREE II quality scores of the guidelines concerning hip replacement. Panel (**b**) AGREE II quality scores of the guidelines concerning knee replacement. Panel (**c**) AGREE II quality scores of the guidelines concerning joint replacement in osteoarthritis. Domain 1: scope and purpose, domain 2: stakeholder involvement, domain 3: rigour of development, domain 4: clarity of presentation, domain 5: applicability, domain 6: editorial independence

The quality of the guidelines differed considerably between the AGREE-II domains and the guidelines (Fig. 1). The ranges of the scaled domain scores were: scope and purpose 0.06-0.81, stakeholder involvement 0.19-0.75, rigour of development 0.03-0.88, clarity of presentation 0.33-0.89, applicability 0-0.50, editorial independence 0-0.96. Low scores were frequently attained in the editorial independence domain due to no clear statement on the influence of the funding body and competing interests. In addition, low scores were often attained in the applicability domain, due to no clear statements on monitoring/auditing criteria of the guideline or facilitators and barriers to the application of the guideline. The OARSI and NOV guidelines attained the highest overall scores, 4.78 and 4.46 respectively. This is explained because both guidelines were developed according to the AGREE-II. The lowest scores were attained by the NZ guidelines, THA (0.84) and TKA (0.46). These guidelines primarily consisted of a, from the BOA guidelines derived, summary of statements concerning THA/TKA but limited information on the required 6 domains.

Although the process of guideline development and quality of reporting differed considerably between the guidelines, the given indication criteria for primary THA and TKA are similar across guidelines (pain, function, radiological changes). Hence, it seems that guideline quality did not influence the main domains included in the indication sets.

### Publications

**Fig. 2:**
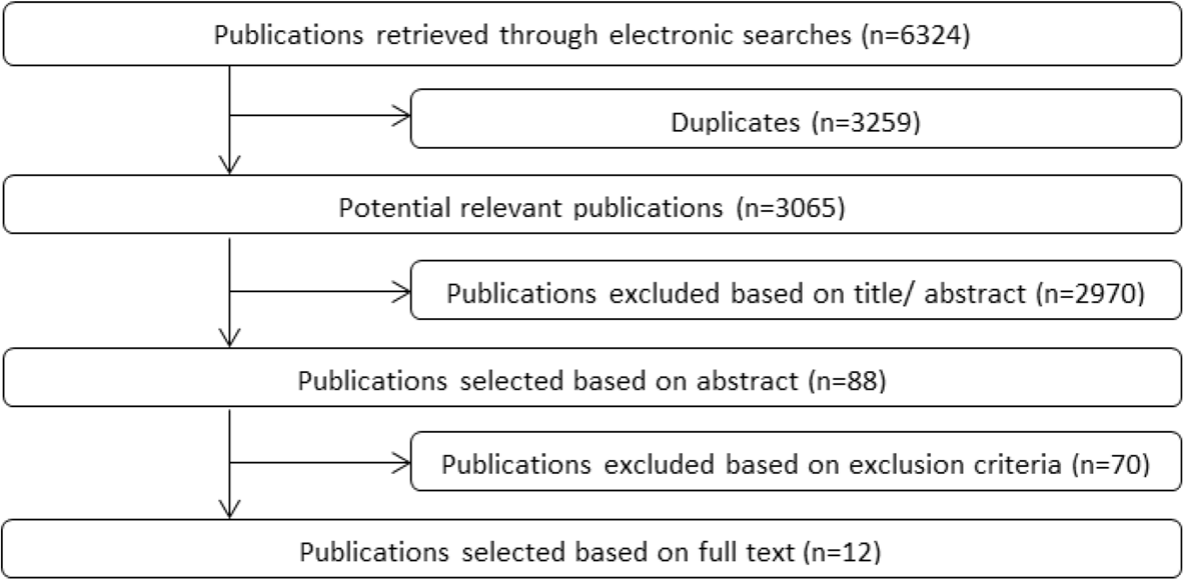
Flow diagram

Our literature search yielded 3065 references (Fig. 2), the full-text of 88 papers was assessed on eligibility. Of these 70 were excluded mainly because no indication criteria for THA/TKA in OA patients were reported. Finally, 18 papers were included (12 reviews/6 original studies).

#### Reviews

Of the included 12 reviews, only 2 were systematic reviews (Table 2) [25, 26]. Furthermore, only 2 reviews focussed on indications for THA/TKA as their main topic [27, 28]. In addition, 1 review investigated the indications for THA/TKA referral [29]. Other topics on which the reviews focussed were management of THA/TKA [30–32], effectiveness of THA/TKA [26] and state of the art overviews of THA/TKA [14, 33, 34].

**Table 2:** Reviews on indication criteria concerning total hip arthroplasty and total knee arthroplasty

Author	Year of publication	Study group region	Systematic review	Evidence	Indication criteria				
					Pain	Function	Radiological changes	Failed or futile conservative therapy	Other criteria
Knee									
Hanssen [34]	2000	USA	No	Level IV					As the indications continue to expand, the decision to proceed with total knee arthroplasty in young, active patients’ needs to be individualized after careful consideration of alternatives.
Kirschner [44]	2011	Germany	No	Level IV	Pain during activities or rest		Radiologic evidence of arthritis		
van Manen [30]	2012	USA	No	Level IV	Severe, refractory knee pain, often at night	Difficulty with activities of daily living; decreased mobility	Radiographic evidence of primary or inflammatory degenerative joint disease: Narrowed joint space; osteophytes (spurring) and bone cysts; squaring of condyles; bone sclerosis	Failure to respond to conservative measures	Current health status: Medically optimized for surgery; no evidence of infection; intact extensor mechanism; informed consent obtained
Medical Advisory Secretariat [26]	2005	Canada	Yes	Level IV	Pain	Functional ability			
Schneppenheim [28]	2001	Germany	No	Level IV	Debilitating pain	Severe restrictions on the activities of the patients in daily life	Significant radiographic findings	Yes	
Hip									
Kirschner [43, 44]	2011	Germany	No	Level IV	Hip: pain during activities or rest	Constricted range of motion	Radiologic evidence of arthritis		
Lane [33]	2007	USA	No	Level IV		Substantial functional impairment			Chronic discomfort
Levine [31]	2013	USA	No	Level IV	Pain refractory to nonsurgical management	Functional impairment	Radiographic findings (joint space narrowing, bone sclerosis, bone cysts femoral/ acetabular osteophytes	Yes	Physical exam findings (groin pain and decreased internal rotation), ruled out causes of referred pain including spine problems and bursitis
Passias [32]^a^	2006	USA	No	Level IV	Incapacitating pain not responsive to conservative therapy	Function limiting symptoms: a significant deterioration in the ability to perform certain activities that are deemed important to the patient, and major lifestyle changes	Evidence of joint degeneration	Yes	
Pivec [14]	2012	USA	No	Level IV	Pain	Functional impairment	Radiographic findings		Initial course of conservative therapy should always be attempted with analgesia, activity modification, ambulatory aids, and weight loss.
Knee and Hip									
Altman [25]	2005	USA, France, Portugal, Belgium, Spain, Germany, Austria, Czech Republic, The Netherlands	Yes	Level III					The criteria for when to perform such surgery are not clear.
Dowsey [27]	2014	Australia	No	Level III					Selection of suitable candidates for TJA is critical but appropriate criteria are not clearly defined.
Mandl [29]	2013	USA	No	Level IV					There are no definitive recommendations for deciding which patients should be referred for TJA.

Legend: TJA: total joint arthroplasty

^a^This study focusses on THA in older people (>65 years of age)

Pain not responsive to conservative treatment, in patients who have functional limitations and radiographic evidence of joint degeneration was most often reported as THA/TKA indication (Table 2). No specific cut-off values were mentioned. It was often not stated if deviations in all these domains should be apparent, or which combinations should be apparent to indicate THA or TKA. Furthermore, the evidence behind all these indication criteria was very low (level IV). In 3 of the reviews the experts explicitly stated that no appropriate indication sets are available for performing THA/TKA.

#### Original publications

Three original publications reported on TKA [8, 9, 35] and 3 on THA [10].

Yamabe et al. [35] considered severe cartilage defects as an optimal indication for TKA. In their discussion section they also included pain but no referral was made to any evidence or the way these indications were established.

The other 5 included original studies investigated decision tools to assess the appropriateness of TKA (*n* = 2) [8, 9] or THA (*n* = 3) [10, 11, 36] in OA patients.

#### TKA appropriateness

Two studies evaluated algorithms to assess TKA appropriateness [8, 9]. The Escobar algorithm was established using the RAND/UCLA appropriateness method, in which expert opinion is combined with available scientific evidence [37]. The following variables where taken into account in different combinations: symptomatology, radiology, age, mobility and stability, previous surgical management and localization. Symptomatology and radiology were the largest contributors in explaining the variability of appropriateness in their model. Table 3 depicts various scenarios in which TKA was considered inappropriate, uncertain or appropriate [9]. However, appropriateness was rated uncertain in a high percentage of scenarios (24.5 %). Another study showed that patients with their TKA rated as appropriate were more likely to achieve better health-related quality of life than patients for whom the TKA was rated as inappropriate [38].

**Table 3:** Different scenarios in which TKA is deemed appropriate, uncertain or inappropriate according to Escobar et al. [9]

Symptoms	Radiology	Age	Mobility	Localisation	Total knee arthroplasty
Slight or moderate	Ahlbäck I-III				Inappropriate
Slight	Ahlbäck IV-V				Inappropriate
Moderate	Ahlbäck IV-V	<55			Inappropriate
Moderate	Ahlbäck IV-V	≥55		Uni	Inappropriate
Moderate	Ahlbäck IV-V	≥55		Bi-tri	Appropriate
Intense-severe	Ahlbäck I-III	<55		Uni-bi	Inappropriate
Intense-severe	Ahlbäck I-III	<55		Tri	Uncertain
Intense-severe	Ahlbäck I	≥55	Normal		Inappropriate
Intense-severe	Ahlbäck II-III	≥55	Normal		Uncertain
Intense-severe	Ahlbäck I	55-65	Limited		Uncertain
Intense	Ahlbäck I	>65	Limited		Uncertain
Severe	Ahlbäck I	>65	Limited		Appropriate
Intense-severe	Ahlbäck II-III	≥55	Limited		Appropriate
Intense-severe	Ahlbäck IV-V	<55		Uni	Uncertain
Intense-severe	Ahlbäck IV-V	<55		Bi-tri	Appropriate
Intense-severe	Ahlbäck IV-V	≥55			Appropriate

Legend: uni: unicompartmental excluded patello-femoral isolated; bi: unicompartmental plus patello-femoral; tri: tricompartmental

Riddle et al. modified the Escobar algorithm to attain a decision tool for US patients [8]. They used the Kellgren Lawrence score rather than the Ahlbäck classification and quantified symptomatology using the Western Ontario and McMaster osteoarthritis index (WOMAC). In 21.7 % of patients appropriateness of TKA was rated as uncertain.

#### THA appropriateness

Quintana et al. developed three THA appropriateness algorithms in OA patients [10, 11, 36]. Two were established using the RAND/UCLA appropriateness method. These algorithms took the following variables into account: age, surgical risk, previous nonsurgical treatments, pain and functional limitation. Table 4 depicts various scenarios in which THA was considered inappropriate, uncertain or appropriate [10]. In both algorithms, appropriateness was rated uncertain in a large part of patients, 46.2 % and 32.4 %. Both algorithms were validated in a population of OA patients scheduled for THA [10, 11]. Patients rated as appropriate THA candidates had better outcomes at 3 months on the WOMAC stiffness and functional limitation domains compared to inappropriate candidates.

**Table 4:** Different scenarios in which THA is deemed appropriate, uncertain or inappropriate according to Quintana et al. [10]

Pain	Non-surgical procedure	Functional limitation	Surgical risk	Age	Total hip arthroplasty
Severe	Correctly	Severe			Appropriate
Severe	Correctly	Minor or moderate			Appropriate
Severe	Not done or not done correctly	Severe			Appropriate
Mild or moderate	Correctly	Severe	Low		Appropriate
Mild		Minor			Inappropriate
Mild		Moderate	High		Inappropriate
Mild		Moderate	Low		Inappropriate
Moderate or severe	Not done or not done correctly			<50 years	Inappropriate
Moderate or severe	Not done or not done Correctly	Minor		>50 years	Inappropriate
Mild or moderate	Not done or not done correctly	Severe	Low		Uncertain
Mild or moderate	Not done or not done correctly	Severe	High		Uncertain
Mild or moderate	Correctly	Severe	High		Uncertain
Severe	Not done or not done correctly	Minor or moderate			Uncertain
Moderate	Correctly	Minor or moderate	High		Uncertain
Moderate	Correctly	Minor or moderate	Low		Uncertain
Moderate	Not done or not done correctly	Moderate		>50	Uncertain
Mild	Correctly	Moderate	Low		Uncertain

The other algorithm was based on the WOMAC as they wanted to develop a tool based on a disease specific instrument rather than on expert opinion [36]. Surgical risk, pre-intervention pain and functional limitations were found to significantly predict changes in the WOMAC pain domain 6 months after THA and pre-intervention functional limitations predicted changes in the functional limitation domain [36]. In addition, by means of a classification and regression tree analysis a summary tree was constructed. THA was rated as appropriate when pain was qualified as severe (according to the pain and limitation short scales), when WOMAC pain pre-intervention score was >60 or when WOMAC functional limitation pre-intervention was >60 with pain pre-intervention >40. Surgical risk was not included in the decision tree. However, the authors stated that one should be aware that higher surgical risk often results in a worse outcome and that conservative treatment should always be performed before considering THA. Again this decision tool was validated in a THA cohort. They assessed sensitivity and specificity of being classified as appropriate compared with the appropriateness based on the minimal clinical important difference values (gain in WOMAC 6 months after THA, pain domain ≥30, function domain ≥25). A sensitivity of 95.0 % and a specificity of 41.0 % were found, suggesting that it seems difficult to identify the non-appropriate cases.

## Discussion

In this systematic review we examined the quality and evidence base of existing indication criteria and guidelines for primary THA and TKA in OA patients. Across guidelines and publications we found, 12 THA, 10 TKA and 2 THA/TKA indication sets. Only 6 guidelines included indication criteria for THA/TKA with differing quality. Overall quality of the guidelines summed across the 6 domains ranged from 0.46 to 4.78. Low scores were frequently attained in the editorial independence domain and the applicability domain. High scores were often attained in the clarity of presentation domain. In the additional 12 reviews and 6 original publications most indication criteria included the following three domains: pain, function and radiological changes. Frequently a prerequisite was that conservative treatment had been insufficient in controlling pain. However, domain specific cut-off values or ranges were mostly not reported. Also, it was often not stated if pain, functional disability and radiological changes should all exist, or which combinations of domain-specific deviations should be apparent to indicate THA or TKA. The level of evidence was low (level IV).

We were not able to discriminate between high and poor quality guidelines as the AGREE-II has not given a set of rules to define a high quality guideline. Given the low scores in the applicability and the editorial independence domains, we advise guideline developers to pay more attention in reporting these issues. A limitation of the current study may be that the scoring of guidelines according to the AGREE-II is not completely objective, even though the manual clearly articulates how each item should be scored including the criteria and considerations for each item. However, the weighting of criteria and considerations in the overall scoring of the item is not mentioned, which could introduce inter-observer variability. To cope with this, the AGREE-II proposes to use more than one observer, which is why the guidelines were scored independently by two investigators and compared to reach consensus (with or without a decisive vote of a third investigator). As such, we tried to minimalize subjectivity.

Another limitation of this study is that we restricted our search strategy to English, Dutch and German language papers. In addition, our website search of orthopaedic and arthritis organizations was restricted to English and Dutch websites. Therefore we may have missed part of the existing literature on indication criteria for THA/TKA.

Irrespective of the quality of individual guidelines, the same domains concerning THA/TKA indications were reported across most guidelines. Based on the design of included studies, the highest level of evidence was reported by the OARSI and EULAR guidelines (only non-experimental studies, level III evidence). The evidence on which indication sets were based came from studies investigating the effectiveness and safety of THA/TKA, but these studies did not specifically address THA/TKA indication sets. Therefore the evidence from these guidelines was rated as level IV evidence, so that the evidence on which indication criteria are based, is low quality evidence.

Looking at other literature, most of the reviews also did not specifically focus on THA/TKA indications and none of the systematic reviews did. Moreover, in 2 of 3 reviews with THA/TKA indication as the main topic it was concluded that no conclusive evidence on THA/TKA indications are currently available. Furthermore, few original papers investigating THA/TKA indications were found, which may be partly due to the employed language restrictions, possibly resulting in language bias. Four of five original studies came from the same group and four were based on the RAND/UCLA method [8–11]. Although this is a respected approach, the limitation is that the indication set is mainly based on expert opinion if little research is available. Thus, even with an optimal composition of experts in the panel, the level of evidence will still be low. This is currently the case for THA/TKA indication sets. In addition, within the proposed THA/TKA decision tools, the appropriateness of surgery was rated as uncertain in many patients. This makes these decision tools difficult to use in daily practice, as patients with appropriateness rated as uncertain may have similar improvements in health outcomes as patients rated as appropriate. Therefore, no evidence-based indications concerning THA/TKA are currently available which can be uniformly used in daily practice.

Nonetheless, when indications were reported, the same domains were included. Hence, although evidence based studies are lacking, expert opinion seems reasonably consistent. This is promising as these domains may give clues to the targets on which future research for THA/TKA indications should focus. It seems evident that pain, function, radiological changes and failed conservative therapy should be part of future studies on THA/TKA indications. The research of indication criteria is, however, difficult. One of the difficulties is that pain and function are relatively subjective measures both when reported by the patient and when judged by the physician. This is illustrated by the fact that although consensus on the indication domains seems to exist, disease severity greatly varies at the time of surgery across different centres in Europe and Australia [6, 7]. This suggests no agreement on the cut-off values or ranges within these domains or between combinations of domains as an indication for surgery. Another difficulty is that it is not possible to conduct controlled trials with the timing of surgery randomized, so that other designs are needed. As a consequence the highest level of evidence is not likely to be obtained, but likely to be relatively low given mainly observational studies (level II and III). However, outcomes of observational studies can be valid and may provide similar results as RCTs. For instance, meta-analyses comparing RCTs and observational studies of treatment effects found no large systematic differences [39]. Furthermore, randomization will avoid confounding by indication but this can also be achieved with advanced statistical analyses and pseudo-randomization in observational studies. To obtain the best possible evidence, we should try to identify predictors for a (less than) good outcome after THA/TKA. With the identified predictors we might be able to simulate with mathematical modelling at which cut-off points surgery has the best postoperative outcomes, taking into account the limited lifespan of a prosthesis and the fact that revision surgery mostly has worse outcomes than primary surgery.

## Conclusions

In conclusion, our current study gives an overview of the available evidence base of THA/TKA indication criteria in both guidelines and original studies. We showed that the currently available THA/TKA indication criteria are based on limited and low quality evidence. Hence, empirical research on this topic is needed, especially regarding domain specific cut-off values or ranges at which the best postoperative outcomes are achieved.

## Additional file

Additional file 1: Search strategy. (DOCX 31 kb)

## Abbreviations

AGREE-II: Appraisal of Guidelines for Research and Evaluation instrument, Dutch version
OA: Osteoarthritis
THA: Total hip arthroplasty
TKA: Total knee arthroplasty
WOMAC: Western Ontario and McMaster osteoarthritis index
