# Supplementary material for: Indication criteria for total hip or knee arthroplasty in osteoarthritis: a state-of-the-science overview
Source: BMC Musculoskelet Disord. 2016 Nov 9;17:463. doi: 10.1186/s12891-016-1325-z (PMC5103467; doi:10.1186/s12891-016-1325-z)
Supplement: Additional file 1: — Search strategy. (DOCX 31 kb) [file 12891_2016_1325_MOESM1_ESM.docx]

**Supplement 1**

The websites of several orthopaedic associations and arthritis organizations (with English or Dutch websites) were independently searched by two authors for guidelines concerning primary hip/knee replacement for OA : American Academy of Orthopaedic Surgeons, American College of Rheumatology, American Association of Orthopaedic Medicine, American Orthopaedic Association, Asia Pacific Orthopaedic Association, Asia Pacific League of Association for Rheumatology, Australian Orthopaedic Association, Australian Rheumatology Association, British Orthopaedic Association (BOA), British Society for Rheumatology, Canadian Orthopaedic association, Canadian Rheumatology Association, Dutch Society of Rheumatology, the European league against rheumatism (EULAR), European Federation of National Associations of Orthopaedics and Traumatology, European Orthopaedic Research Society, Irish Society of Orthopaedic Medicine, Irish Institute of Trauma and Orthopaedic Surgery, Irish Society for Rheumatology, Indian Rheumatology Association, Indian Orthopaedic Association, National Institute for Health Care Excellence, Netherlands Orthopaedic Association (NOV), New Zealand Orthopaedic Association (NZ), Nordic orthopaedic association, National Institute for Health and Care Excellence (NICE), New Zealand Rheumatology Association, Osteoarthritis Research Society International (OARSI), South African League Against Rheumatism and Arthritis Organization and South African Orthopaedic Association.

The search strategy for identification of publications on indications for THA and TKA is depicted in the table here below.

| **Databases** | Search Strategy | Number unique references |
| --- | --- | --- |
|  |  |  |
| PubMed | **(**("Arthroplasty, Replacement, Knee"[majr] OR "Knee Prosthesis"[majr] OR "knee replacement arthroplasty"[ti] OR "total knee arthroplasty"[ti] OR "total knee"[ti] OR tka[ti] OR "tkr"[ti] OR "total knee replacement"[ti] OR "knee prosthesis"[ti] OR "knee implantation"[ti] OR "knee implant"[ti] OR "knee implants"[ti] OR "knee prosthesis"[ti] OR "knee joint replacement"[ti] OR "knee joint arthroplasty"[ti] OR "Knee Replacement Arthroplasties"[ti] OR "Total Knee Replacements"[ti] OR "Knee Prostheses"[ti] OR "Knee endoprosthesis"[ti] OR "Knee endoprostheses"[ti] OR "Knee joint arthroplasty"[ti] OR "Knee joint arthroplasties"[ti] OR "knee joint prosthesis"[ti] OR "knee joint prostheses"[ti] OR "knee prosthetic"[ti] OR "Knee endoprosthetic"[ti] OR "knee joint prosthetic"[ti] OR "Knee joint endoprosthetic"[ti] OR "knee prosthetics"[ti] OR "Knee endoprosthetics"[ti] OR "knee joint prosthetics"[ti] OR "Knee joint endoprosthetics"[ti] OR "Knee replacement"[ti] OR "Knee replacements"[ti] OR "knee arthroplasty"[ti] OR "knee arthroplasties"[ti] **OR** "Arthroplasty, Replacement, Hip"[majr] OR "Hip Prosthesis"[majr] OR "hip replacement arthroplasty"[ti] OR "total hip arthroplasty"[ti] OR "total hip"[ti] OR tha[ti] OR "thr"[ti] OR "total hip replacement"[ti] OR "hip prosthesis"[ti] OR "hip implantation"[ti] OR "hip implant"[ti] OR "hip implants"[ti] OR "hip prosthesis"[ti] OR "hip joint replacement"[ti] OR "hip joint arthroplasty"[ti] OR "Hip Replacement Arthroplasties"[ti] OR "Total Hip Replacements"[ti] OR "Hip Prostheses"[ti] OR "Hip endoprosthesis"[ti] OR "Hip endoprostheses"[ti] OR "Hip joint arthroplasty"[ti] OR "Hip joint arthroplasties"[ti] OR "hip joint prosthesis"[ti] OR "hip joint prostheses"[ti] OR "hip prosthetic"[ti] OR "Hip endoprosthetic"[ti] OR "hip joint prosthetic"[ti] OR "Hip joint endoprosthetic"[ti] OR "hip prosthetics"[ti] OR "Hip endoprosthetics"[ti] OR "hip joint prosthetics"[ti] OR "Hip joint endoprosthetics"[ti] OR "Hip replacement"[ti] OR "Hip replacements"[ti] OR "hip arthroplasty"[ti] OR "hip arthroplasties"[ti] **OR (("total joint arthroplasty"[ti] OR "total joint replacement"[ti] OR "total joint prosthesis"[ti] OR "Arthroplasty, Replacement"[Majr:NoExp]**) NOT (shoulder*[ti] OR ankle*[ti]))) AND ((decision[ti] AND "to operate"[ti]) OR "decision to treat"[ti] OR "treatment decision"[ti] OR "operation decision"[ti] OR "surgery decision"[ti] OR "intervention decision"[ti] OR "treatment decisions"[ti] OR "operation decisions"[ti] OR "surgery decisions"[ti] OR "intervention decisions"[ti] OR "Patient Selection"[Mesh] OR "patient selection"[ti] OR "Patient Selections"[ti] OR "Selection for Treatment"[ti] OR "Selection for Treatments"[ti] OR "Selection of Subjects"[ti] OR "Subjects Selection"[ti] OR "Subjects Selections"[ti] OR "Selection Criteria"[ti] OR "priority tool"[ti] OR "priority tools"[ti] OR "priority criteria"[ti] OR "priority criterium"[ti] OR "indication set"[ti] OR "priority"[ti] OR "priorities"[ti] OR priorit*[ti] OR "indication"[ti] OR "indications"[ti] OR "**appropriateness criteria"[ti] OR ("evidence"[tw] AND ("indication"[tw] OR "indications"[tw])) OR (("Checklist"[Mesh] OR "checklist"[tw] OR "checklists"[tw]) AND ("indication"[tw] OR "indications"[tw])) OR "Guideline"[Publication Type] OR "Practice Guideline"[Publication Type] OR "Guidelines as Topic"[majr] OR "Practice Guidelines as Topic"[majr] OR (("guideline"[ti] OR "guidelines"[ti]) NOT "Guidelines for Authors"))) OR (**("Osteoarthritis"[Mesh] OR "Osteoarthritis"[tw] OR "Osteoarthritides"[tw] OR "Osteoarthrosis"[tw] OR "Osteoarthroses"[tw] OR "Degenerative Arthritides"[tw] OR "Degenerative Arthritis"[tw] OR OA[ti]) AND ("Arthroplasty, Replacement, Knee"[majr] OR "Knee Prosthesis"[majr] OR "knee replacement arthroplasty"[ti] OR "total knee arthroplasty"[ti] OR "total knee"[ti] OR tka[ti] OR "tkr"[ti] OR "total knee replacement"[ti] OR "knee prosthesis"[ti] OR "knee implantation"[ti] OR "knee implant"[ti] OR "knee implants"[ti] OR "knee prosthesis"[ti] OR "knee joint replacement"[ti] OR "knee joint arthroplasty"[ti] OR "Knee Replacement Arthroplasties"[ti] OR "Total Knee Replacements"[ti] OR "Knee Prostheses"[ti] OR "Knee endoprosthesis"[ti] OR "Knee endoprostheses"[ti] OR "Knee joint arthroplasty"[ti] OR "Knee joint arthroplasties"[ti] OR "knee joint prosthesis"[ti] OR "knee joint prostheses"[ti] OR "knee prosthetic"[ti] OR "Knee endoprosthetic"[ti] OR "knee joint prosthetic"[ti] OR "Knee joint endoprosthetic"[ti] OR "knee prosthetics"[ti] OR "Knee endoprosthetics"[ti] OR "knee joint prosthetics"[ti] OR "Knee joint endoprosthetics"[ti] OR "Knee replacement"[ti] OR "Knee replacements"[ti] OR "knee arthroplasty"[ti] OR "knee arthroplasties"[ti] **OR** "Arthroplasty, Replacement, Hip"[majr] OR "Hip Prosthesis"[majr] OR "hip replacement arthroplasty"[ti] OR "total hip arthroplasty"[ti] OR "total hip"[ti] OR tha[ti] OR "thr"[ti] OR "total hip replacement"[ti] OR "hip prosthesis"[ti] OR "hip implantation"[ti] OR "hip implant"[ti] OR "hip implants"[ti] OR "hip prosthesis"[ti] OR "hip joint replacement"[ti] OR "hip joint arthroplasty"[ti] OR "Hip Replacement Arthroplasties"[ti] OR "Total Hip Replacements"[ti] OR "Hip Prostheses"[ti] OR "Hip endoprosthesis"[ti] OR "Hip endoprostheses"[ti] OR "Hip joint arthroplasty"[ti] OR "Hip joint arthroplasties"[ti] OR "hip joint prosthesis"[ti] OR "hip joint prostheses"[ti] OR "hip prosthetic"[ti] OR "Hip endoprosthetic"[ti] OR "hip joint prosthetic"[ti] OR "Hip joint endoprosthetic"[ti] OR "hip prosthetics"[ti] OR "Hip endoprosthetics"[ti] OR "hip joint prosthetics"[ti] OR "Hip joint endoprosthetics"[ti] OR "Hip replacement"[ti] OR "Hip replacements"[ti] OR "hip arthroplasty"[ti] OR "hip arthroplasties"[ti] **OR (("total joint arthroplasty"[ti] OR "total joint replacement"[ti] OR "total joint prosthesis"[ti] OR "Arthroplasty, Replacement"[Majr:NoExp])** NOT (shoulder*[ti] OR ankle*[ti]))) AND ((decision[tw] AND "to operate"[tw]) OR "decision to treat"[tw] OR "treatment decision"[tw] OR "operation decision"[tw] OR "surgery decision"[tw] OR "intervention decision"[tw] OR "treatment decisions"[tw] OR "operation decisions"[tw] OR "surgery decisions"[tw] OR "intervention decisions"[tw] OR "Patient Selection"[Mesh] OR "patient selection"[tw] OR "Patient Selections"[tw] OR "Selection for Treatment"[tw] OR "Selection for Treatments"[tw] OR "Selection of Subjects"[tw] OR "Subjects Selection"[tw] OR "Subjects Selections"[tw] OR "Selection Criteria"[tw] OR "priority tool"[tw] OR "priority tools"[tw] OR "priority criteria"[tw] OR "priority criterium"[tw] OR "indication set"[tw] OR "priority"[tw] OR "priorities"[tw] OR priorit*[tw] OR "indication"[tw] OR "indications"[tw] OR "**appropriateness criteria"[tw] OR ("evidence"[tw] AND ("indication"[tw] OR "indications"[tw])) OR (("Checklist"[Mesh] OR "checklist"[tw] OR "checklists"[tw]) AND ("indication"[tw] OR "indications"[tw])) OR "Guideline"[Publication Type] OR "Practice Guideline"[Publication Type] OR "Guidelines as Topic"[Mesh] OR "Practice Guidelines as Topic"[Mesh] OR (("guideline"[tw] OR "guidelines"[tw]) NOT "Guidelines for Authors")))** | 1516 |
|  |  |  |
| MEDLINE (OVID-version) | **(**(exp *Arthroplasty, Replacement, Knee/ OR exp *Knee Prosthesis/ OR "knee replacement arthroplasty".ti OR "total knee arthroplasty".ti OR "total knee".ti OR tka.ti OR "tkr".ti OR "total knee replacement".ti OR "knee prosthesis".ti OR "knee implantation".ti OR "knee implant".ti OR "knee implants".ti OR "knee prosthesis".ti OR "knee joint replacement".ti OR "knee joint arthroplasty".ti OR "Knee Replacement Arthroplasties".ti OR "Total Knee Replacements".ti OR "Knee Prostheses".ti OR "Knee endoprosthesis".ti OR "Knee endoprostheses".ti OR "Knee joint arthroplasty".ti OR "Knee joint arthroplasties".ti OR "knee joint prosthesis".ti OR "knee joint prostheses".ti OR "knee prosthetic".ti OR "Knee endoprosthetic".ti OR "knee joint prosthetic".ti OR "Knee joint endoprosthetic".ti OR "knee prosthetics".ti OR "Knee endoprosthetics".ti OR "knee joint prosthetics".ti OR "Knee joint endoprosthetics".ti OR "Knee replacement".ti OR "Knee replacements".ti OR "knee arthroplasty".ti OR "knee arthroplasties".ti **OR** exp *Arthroplasty, Replacement, Hip/ OR exp *Hip Prosthesis/ OR "hip replacement arthroplasty".ti OR "total hip arthroplasty".ti OR "total hip".ti OR tha.ti OR "thr".ti OR "total hip replacement".ti OR "hip prosthesis".ti OR "hip implantation".ti OR "hip implant".ti OR "hip implants".ti OR "hip prosthesis".ti OR "hip joint replacement".ti OR "hip joint arthroplasty".ti OR "Hip Replacement Arthroplasties".ti OR "Total Hip Replacements".ti OR "Hip Prostheses".ti OR "Hip endoprosthesis".ti OR "Hip endoprostheses".ti OR "Hip joint arthroplasty".ti OR "Hip joint arthroplasties".ti OR "hip joint prosthesis".ti OR "hip joint prostheses".ti OR "hip prosthetic".ti OR "Hip endoprosthetic".ti OR "hip joint prosthetic".ti OR "Hip joint endoprosthetic".ti OR "hip prosthetics".ti OR "Hip endoprosthetics".ti OR "hip joint prosthetics".ti OR "Hip joint endoprosthetics".ti OR "Hip replacement".ti OR "Hip replacements".ti OR "hip arthroplasty".ti OR "hip arthroplasties".ti **OR (("total joint arthroplasty".ti OR "total joint replacement".ti OR "total joint prosthesis".ti OR *Arthroplasty, Replacement/**) NOT (shoulder*.ti OR ankle*.ti))) AND ((decision.ti AND "to operate".ti) OR "decision to operate".ti OR "decision to treat".ti OR "treatment decision".ti OR "operation decision".ti OR "surgery decision".ti OR "intervention decision".ti OR "treatment decisions".ti OR "operation decisions".ti OR "surgery decisions".ti OR "intervention decisions".ti OR exp Patient Selection/ OR "patient selection".ti OR "Patient Selections".ti OR "Selection for Treatment".ti OR "Selection for Treatments".ti OR "Selection of Subjects".ti OR "Subjects Selection".ti OR "Subjects Selections".ti OR "Selection Criteria".ti OR "priority tool".ti OR "priority tools".ti OR "priority criteria".ti OR "priority criterium".ti OR "indication set".ti OR "priority".ti OR "priorities".ti OR priorit*.ti OR "indication".ti OR "indications".ti OR "**appropriateness criteria".ti OR ("evidence".mp AND ("indication".mp OR "indications".mp)) OR ((Checklist/ OR "checklist".mp OR "checklists".mp) AND ("indication".mp OR "indications".mp)) OR exp Guideline/ OR exp Practice Guideline/ OR exp *"Guidelines as Topic"/ OR exp *"Practice Guidelines as Topic"/ OR (("guideline".ti OR "guidelines".ti) NOT "Guidelines for Authors".ti,ab))) OR (**(exp Osteoarthritis/ OR "Osteoarthritis".mp OR "Osteoarthritides".mp OR "Osteoarthrosis".mp OR "Osteoarthroses".mp OR "Degenerative Arthritides".mp OR "Degenerative Arthritis".mp OR OA.ti) AND (exp *Arthroplasty, Replacement, Knee/ OR exp *Knee Prosthesis/ OR "knee replacement arthroplasty".ti OR "total knee arthroplasty".ti OR "total knee".ti OR tka.ti OR "tkr".ti OR "total knee replacement".ti OR "knee prosthesis".ti OR "knee implantation".ti OR "knee implant".ti OR "knee implants".ti OR "knee prosthesis".ti OR "knee joint replacement".ti OR "knee joint arthroplasty".ti OR "Knee Replacement Arthroplasties".ti OR "Total Knee Replacements".ti OR "Knee Prostheses".ti OR "Knee endoprosthesis".ti OR "Knee endoprostheses".ti OR "Knee joint arthroplasty".ti OR "Knee joint arthroplasties".ti OR "knee joint prosthesis".ti OR "knee joint prostheses".ti OR "knee prosthetic".ti OR "Knee endoprosthetic".ti OR "knee joint prosthetic".ti OR "Knee joint endoprosthetic".ti OR "knee prosthetics".ti OR "Knee endoprosthetics".ti OR "knee joint prosthetics".ti OR "Knee joint endoprosthetics".ti OR "Knee replacement".ti OR "Knee replacements".ti OR "knee arthroplasty".ti OR "knee arthroplasties".ti **OR** exp *Arthroplasty, Replacement, Hip/ OR exp *Hip Prosthesis/ OR "hip replacement arthroplasty".ti OR "total hip arthroplasty".ti OR "total hip".ti OR tha.ti OR "thr".ti OR "total hip replacement".ti OR "hip prosthesis".ti OR "hip implantation".ti OR "hip implant".ti OR "hip implants".ti OR "hip prosthesis".ti OR "hip joint replacement".ti OR "hip joint arthroplasty".ti OR "Hip Replacement Arthroplasties".ti OR "Total Hip Replacements".ti OR "Hip Prostheses".ti OR "Hip endoprosthesis".ti OR "Hip endoprostheses".ti OR "Hip joint arthroplasty".ti OR "Hip joint arthroplasties".ti OR "hip joint prosthesis".ti OR "hip joint prostheses".ti OR "hip prosthetic".ti OR "Hip endoprosthetic".ti OR "hip joint prosthetic".ti OR "Hip joint endoprosthetic".ti OR "hip prosthetics".ti OR "Hip endoprosthetics".ti OR "hip joint prosthetics".ti OR "Hip joint endoprosthetics".ti OR "Hip replacement".ti OR "Hip replacements".ti OR "hip arthroplasty".ti OR "hip arthroplasties".ti **OR (("total joint arthroplasty".ti OR "total joint replacement".ti OR "total joint prosthesis".ti OR *Arthroplasty, Replacement/)** NOT (shoulder*.ti OR ankle*.ti))) AND ((decision.mp AND "to operate".mp) OR "decision to operate".mp OR "decision to treat".mp OR "treatment decision".mp OR "operation decision".mp OR "surgery decision".mp OR "intervention decision".mp OR "treatment decisions".mp OR "operation decisions".mp OR "surgery decisions".mp OR "intervention decisions".mp OR exp Patient Selection/ OR "patient selection".mp OR "Patient Selections".mp OR "Selection for Treatment".mp OR "Selection for Treatments".mp OR "Selection of Subjects".mp OR "Subjects Selection".mp OR "Subjects Selections".mp OR "Selection Criteria".mp OR "priority tool".mp OR "priority tools".mp OR "priority criteria".mp OR "priority criterium".mp OR "indication set".mp OR "priority".mp OR "priorities".mp OR priorit*.mp OR "indication".mp OR "indications".mp OR "**appropriateness criteria".mp OR ("evidence".mp AND ("indication".mp OR "indications".mp)) OR ((Checklist/ OR "checklist".mp OR "checklists".mp) AND ("indication".mp OR "indications".mp)) OR exp Guideline/ OR exp Practice Guideline/ OR exp "Guidelines as Topic"/ OR exp "Practice Guidelines as Topic"/ OR (("guideline".mp OR "guidelines".mp) NOT "Guidelines for Authors".mp)))** | 4 |
|  |  |  |
| Embase (OVID-version) | (exp *Knee Arthroplasty/ OR exp *Knee Prosthesis/ OR "knee replacement arthroplasty".ti OR "total knee arthroplasty".ti OR "total knee".ti OR tka.ti OR "tkr".ti OR "total knee replacement".ti OR "knee prosthesis".ti OR "knee implantation".ti OR "knee implant".ti OR "knee implants".ti OR "knee prosthesis".ti OR "knee joint replacement".ti OR "knee joint arthroplasty".ti OR "Knee Replacement Arthroplasties".ti OR "Total Knee Replacements".ti OR "Knee Prostheses".ti OR "Knee endoprosthesis".ti OR "Knee endoprostheses".ti OR "Knee joint arthroplasty".ti OR "Knee joint arthroplasties".ti OR "knee joint prosthesis".ti OR "knee joint prostheses".ti OR "knee prosthetic".ti OR "Knee endoprosthetic".ti OR "knee joint prosthetic".ti OR "Knee joint endoprosthetic".ti OR "knee prosthetics".ti OR "Knee endoprosthetics".ti OR "knee joint prosthetics".ti OR "Knee joint endoprosthetics".ti OR "Knee replacement".ti OR "Knee replacements".ti OR "knee arthroplasty".ti OR "knee arthroplasties".ti **OR** exp *Hip Arthroplasty/ OR exp *Hip Prosthesis/ OR "hip replacement arthroplasty".ti OR "total hip arthroplasty".ti OR "total hip".ti OR tha.ti OR "thr".ti OR "total hip replacement".ti OR "hip prosthesis".ti OR "hip implantation".ti OR "hip implant".ti OR "hip implants".ti OR "hip prosthesis".ti OR "hip joint replacement".ti OR "hip joint arthroplasty".ti OR "Hip Replacement Arthroplasties".ti OR "Total Hip Replacements".ti OR "Hip Prostheses".ti OR "Hip endoprosthesis".ti OR "Hip endoprostheses".ti OR "Hip joint arthroplasty".ti OR "Hip joint arthroplasties".ti OR "hip joint prosthesis".ti OR "hip joint prostheses".ti OR "hip prosthetic".ti OR "Hip endoprosthetic".ti OR "hip joint prosthetic".ti OR "Hip joint endoprosthetic".ti OR "hip prosthetics".ti OR "Hip endoprosthetics".ti OR "hip joint prosthetics".ti OR "Hip joint endoprosthetics".ti OR "Hip replacement".ti OR "Hip replacements".ti OR "hip arthroplasty".ti OR "hip arthroplasties".ti) AND ("decision to operate".ti OR "decision to treat".ti OR "treatment decision".ti OR "operation decision".ti OR "surgery decision".ti OR "intervention decision".ti OR "treatment decisions".ti OR "operation decisions".ti OR "surgery decisions".ti OR "intervention decisions".ti OR exp Patient Selection/ OR "patient selection".ti OR "Patient Selections".ti OR "Selection for Treatment".ti OR "Selection for Treatments".ti OR "Selection of Subjects".ti OR "Subjects Selection".ti OR "Subjects Selections".ti OR "Selection Criteria".ti OR "priority tool".ti OR "priority tools".ti OR "priority criteria".ti OR "priority criterium".ti OR "indication set".ti OR "priority".ti OR "priorities".ti OR priorit*.ti OR "indication".ti OR "indications".ti OR * treatment indication/ OR "**appropriateness criteria".ti OR ("evidence".ti AND ("indication".ti OR "indications".ti)) OR ((Checklist/ OR "checklist".ti OR "checklists".ti) AND ("indication".ti OR "indications".ti)) OR exp *practice guideline/ OR (("guideline".ti OR "guidelines".ti) NOT "guidelines for authors".mp))) OR (**(exp Osteoarthritis/ OR "Osteoarthritis".ti,ab OR "Osteoarthritides".ti,ab OR "Osteoarthrosis".ti,ab OR "Osteoarthroses".ti,ab OR "Degenerative Arthritides".ti,ab OR "Degenerative Arthritis".ti,ab OR OA.ti) AND (exp *Knee Arthroplasty/ OR exp *Knee Prosthesis/ OR "knee replacement arthroplasty".ti OR "total knee arthroplasty".ti OR "total knee".ti OR tka.ti OR "tkr".ti OR "total knee replacement".ti OR "knee prosthesis".ti OR "knee implantation".ti OR "knee implant".ti OR "knee implants".ti OR "knee prosthesis".ti OR "knee joint replacement".ti OR "knee joint arthroplasty".ti OR "Knee Replacement Arthroplasties".ti OR "Total Knee Replacements".ti OR "Knee Prostheses".ti OR "Knee endoprosthesis".ti OR "Knee endoprostheses".ti OR "Knee joint arthroplasty".ti OR "Knee joint arthroplasties".ti OR "knee joint prosthesis".ti OR "knee joint prostheses".ti OR "knee prosthetic".ti OR "Knee endoprosthetic".ti OR "knee joint prosthetic".ti OR "Knee joint endoprosthetic".ti OR "knee prosthetics".ti OR "Knee endoprosthetics".ti OR "knee joint prosthetics".ti OR "Knee joint endoprosthetics".ti OR "Knee replacement".ti OR "Knee replacements".ti OR "knee arthroplasty".ti OR "knee arthroplasties".ti **OR** exp *Hip Arthroplasty/ OR exp *Hip Prosthesis/ OR "hip replacement arthroplasty".ti OR "total hip arthroplasty".ti OR "total hip".ti OR tha.ti OR "thr".ti OR "total hip replacement".ti OR "hip prosthesis".ti OR "hip implantation".ti OR "hip implant".ti OR "hip implants".ti OR "hip prosthesis".ti OR "hip joint replacement".ti OR "hip joint arthroplasty".ti OR "Hip Replacement Arthroplasties".ti OR "Total Hip Replacements".ti OR "Hip Prostheses".ti OR "Hip endoprosthesis".ti OR "Hip endoprostheses".ti OR "Hip joint arthroplasty".ti OR "Hip joint arthroplasties".ti OR "hip joint prosthesis".ti OR "hip joint prostheses".ti OR "hip prosthetic".ti OR "Hip endoprosthetic".ti OR "hip joint prosthetic".ti OR "Hip joint endoprosthetic".ti OR "hip prosthetics".ti OR "Hip endoprosthetics".ti OR "hip joint prosthetics".ti OR "Hip joint endoprosthetics".ti OR "Hip replacement".ti OR "Hip replacements".ti OR "hip arthroplasty".ti OR "hip arthroplasties".ti) AND ("decision to operate".ti,ab OR "decision to treat".ti,ab OR "treatment decision".ti,ab OR "operation decision".ti,ab OR "surgery decision".ti,ab OR "intervention decision".ti,ab OR "treatment decisions".ti,ab OR "operation decisions".ti,ab OR "surgery decisions".ti,ab OR "intervention decisions".ti,ab OR exp Patient Selection/ OR "patient selection".ti,ab OR "Patient Selections".ti,ab OR "Selection for Treatment".ti,ab OR "Selection for Treatments".ti,ab OR "Selection of Subjects".ti,ab OR "Subjects Selection".ti,ab OR "Subjects Selections".ti,ab OR "Selection Criteria".ti,ab OR "priority tool".ti,ab OR "priority tools".ti,ab OR "priority criteria".ti,ab OR "priority criterium".ti,ab OR "indication set".ti,ab OR "priority".ti,ab OR "priorities".ti,ab OR priorit*.ti,ab OR "indication".ti,ab OR "indications".ti,ab OR treatment indication/ OR "**appropriateness criteria".ti,ab OR ("evidence".ti,ab AND ("indication".ti,ab OR "indications".ti,ab)) OR ((Checklist/ OR "checklist".ti,ab OR "checklists".ti,ab) AND ("indication".ti,ab OR "indications".ti,ab)) OR exp practice guideline/ OR (("guideline".mp OR "guidelines".mp) NOT "guidelines for authors".mp)))** | 933 |
|  |  |  |
| Web of Science | **TI=(**(Knee Arthroplasty OR Knee Prosthesis OR "knee replacement arthroplasty" OR "total knee arthroplasty" OR "total knee" OR tka OR "tkr" OR "total knee replacement" OR "knee prosthesis" OR "knee implantation" OR "knee implant" OR "knee implants" OR "knee prosthesis" OR "knee joint replacement" OR "knee joint arthroplasty" OR "Knee Replacement Arthroplasties" OR "Total Knee Replacements" OR "Knee Prostheses" OR "Knee endoprosthesis" OR "Knee endoprostheses" OR "Knee joint arthroplasty" OR "Knee joint arthroplasties" OR "knee joint prosthesis" OR "knee joint prostheses" OR "knee prosthetic" OR "Knee endoprosthetic" OR "knee joint prosthetic" OR "Knee joint endoprosthetic" OR "knee prosthetics" OR "Knee endoprosthetics" OR "knee joint prosthetics" OR "Knee joint endoprosthetics" OR "Knee replacement" OR "Knee replacements" OR "knee arthroplasty" OR "knee arthroplasties" **OR** Hip Arthroplasty OR Hip Prosthesis OR "hip replacement arthroplasty" OR "total hip arthroplasty" OR "total hip" OR tha OR "thr" OR "total hip replacement" OR "hip prosthesis" OR "hip implantation" OR "hip implant" OR "hip implants" OR "hip prosthesis" OR "hip joint replacement" OR "hip joint arthroplasty" OR "Hip Replacement Arthroplasties" OR "Total Hip Replacements" OR "Hip Prostheses" OR "Hip endoprosthesis" OR "Hip endoprostheses" OR "Hip joint arthroplasty" OR "Hip joint arthroplasties" OR "hip joint prosthesis" OR "hip joint prostheses" OR "hip prosthetic" OR "Hip endoprosthetic" OR "hip joint prosthetic" OR "Hip joint endoprosthetic" OR "hip prosthetics" OR "Hip endoprosthetics" OR "hip joint prosthetics" OR "Hip joint endoprosthetics" OR "Hip replacement" OR "Hip replacements" OR "hip arthroplasty" OR "hip arthroplasties") AND ("decision to operate" OR "decision to treat" OR "treatment decision" OR "operation decision" OR "surgery decision" OR "intervention decision" OR "treatment decisions" OR "operation decisions" OR "surgery decisions" OR "intervention decisions" OR Patient Selection OR "patient selection" OR "Patient Selections" OR "Selection for Treatment" OR "Selection for Treatments" OR "Selection of Subjects" OR "Subjects Selection" OR "Subjects Selections" OR "Selection Criteria" OR "priority tool" OR "priority tools" OR "priority criteria" OR "priority criterium" OR "indication set" OR "priority" OR "priorities" OR priorit* OR "indication" OR "indications" OR * treatment indication OR "**appropriateness criteria" OR ("evidence" AND ("indication" OR "indications")) OR ((Checklist OR "checklist" OR "checklists") AND ("indication" OR "indications")) OR "guideline" OR "guidelines"))**  OR  **TS=(**(Osteoarthritis OR "Osteoarthritis" OR "Osteoarthritides" OR "Osteoarthrosis" OR "Osteoarthroses" OR "Degenerative Arthritides" OR "Degenerative Arthritis" OR OA) AND (Knee Arthroplasty OR Knee Prosthesis OR "knee replacement arthroplasty" OR "total knee arthroplasty" OR "total knee" OR tka OR "tkr" OR "total knee replacement" OR "knee prosthesis" OR "knee implantation" OR "knee implant" OR "knee implants" OR "knee prosthesis" OR "knee joint replacement" OR "knee joint arthroplasty" OR "Knee Replacement Arthroplasties" OR "Total Knee Replacements" OR "Knee Prostheses" OR "Knee endoprosthesis" OR "Knee endoprostheses" OR "Knee joint arthroplasty" OR "Knee joint arthroplasties" OR "knee joint prosthesis" OR "knee joint prostheses" OR "knee prosthetic" OR "Knee endoprosthetic" OR "knee joint prosthetic" OR "Knee joint endoprosthetic" OR "knee prosthetics" OR "Knee endoprosthetics" OR "knee joint prosthetics" OR "Knee joint endoprosthetics" OR "Knee replacement" OR "Knee replacements" OR "knee arthroplasty" OR "knee arthroplasties" **OR** Hip Arthroplasty OR Hip Prosthesis OR "hip replacement arthroplasty" OR "total hip arthroplasty" OR "total hip" OR tha OR "thr" OR "total hip replacement" OR "hip prosthesis" OR "hip implantation" OR "hip implant" OR "hip implants" OR "hip prosthesis" OR "hip joint replacement" OR "hip joint arthroplasty" OR "Hip Replacement Arthroplasties" OR "Total Hip Replacements" OR "Hip Prostheses" OR "Hip endoprosthesis" OR "Hip endoprostheses" OR "Hip joint arthroplasty" OR "Hip joint arthroplasties" OR "hip joint prosthesis" OR "hip joint prostheses" OR "hip prosthetic" OR "Hip endoprosthetic" OR "hip joint prosthetic" OR "Hip joint endoprosthetic" OR "hip prosthetics" OR "Hip endoprosthetics" OR "hip joint prosthetics" OR "Hip joint endoprosthetics" OR "Hip replacement" OR "Hip replacements" OR "hip arthroplasty" OR "hip arthroplasties") AND ("decision to operate" OR "decision to treat" OR "treatment decision" OR "operation decision" OR "surgery decision" OR "intervention decision" OR "treatment decisions" OR "operation decisions" OR "surgery decisions" OR "intervention decisions" OR Patient Selection OR "patient selection" OR "Patient Selections" OR "Selection for Treatment" OR "Selection for Treatments" OR "Selection of Subjects" OR "Subjects Selection" OR "Subjects Selections" OR "Selection Criteria" OR "priority tool" OR "priority tools" OR "priority criteria" OR "priority criterium" OR "indication set" OR "priority" OR "priorities" OR priorit* OR "indication" OR "indications" OR treatment indication OR "**appropriateness criteria" OR ("evidence" AND ("indication" OR "indications")) OR ((Checklist OR "checklist" OR "checklists") AND ("indication" OR "indications")) OR "guideline" OR "guidelines"))** | 431 |
|  |  |  |
| COCHRANE Library | title  **(**(Knee Arthroplasty OR Knee Prosthesis OR "knee replacement arthroplasty" OR "total knee arthroplasty" OR "total knee" OR tka OR "tkr" OR "total knee replacement" OR "knee prosthesis" OR "knee implantation" OR "knee implant" OR "knee implants" OR "knee prosthesis" OR "knee joint replacement" OR "knee joint arthroplasty" OR "Knee Replacement Arthroplasties" OR "Total Knee Replacements" OR "Knee Prostheses" OR "Knee endoprosthesis" OR "Knee endoprostheses" OR "Knee joint arthroplasty" OR "Knee joint arthroplasties" OR "knee joint prosthesis" OR "knee joint prostheses" OR "knee prosthetic" OR "Knee endoprosthetic" OR "knee joint prosthetic" OR "Knee joint endoprosthetic" OR "knee prosthetics" OR "Knee endoprosthetics" OR "knee joint prosthetics" OR "Knee joint endoprosthetics" OR "Knee replacement" OR "Knee replacements" OR "knee arthroplasty" OR "knee arthroplasties" **OR** Hip Arthroplasty OR Hip Prosthesis OR "hip replacement arthroplasty" OR "total hip arthroplasty" OR "total hip" OR tha OR "thr" OR "total hip replacement" OR "hip prosthesis" OR "hip implantation" OR "hip implant" OR "hip implants" OR "hip prosthesis" OR "hip joint replacement" OR "hip joint arthroplasty" OR "Hip Replacement Arthroplasties" OR "Total Hip Replacements" OR "Hip Prostheses" OR "Hip endoprosthesis" OR "Hip endoprostheses" OR "Hip joint arthroplasty" OR "Hip joint arthroplasties" OR "hip joint prosthesis" OR "hip joint prostheses" OR "hip prosthetic" OR "Hip endoprosthetic" OR "hip joint prosthetic" OR "Hip joint endoprosthetic" OR "hip prosthetics" OR "Hip endoprosthetics" OR "hip joint prosthetics" OR "Hip joint endoprosthetics" OR "Hip replacement" OR "Hip replacements" OR "hip arthroplasty" OR "hip arthroplasties") AND ("decision to operate" OR "decision to treat" OR "treatment decision" OR "operation decision" OR "surgery decision" OR "intervention decision" OR "treatment decisions" OR "operation decisions" OR "surgery decisions" OR "intervention decisions" OR Patient Selection OR "patient selection" OR "Patient Selections" OR "Selection for Treatment" OR "Selection for Treatments" OR "Selection of Subjects" OR "Subjects Selection" OR "Subjects Selections" OR "Selection Criteria" OR "priority tool" OR "priority tools" OR "priority criteria" OR "priority criterium" OR "indication set" OR "priority" OR "priorities" OR priorit* OR "indication" OR "indications" OR * treatment indication OR "**appropriateness criteria" OR ("evidence" AND ("indication" OR "indications")) OR ((Checklist OR "checklist" OR "checklists") AND ("indication" OR "indications"))** **OR "guideline" OR "guidelines"))**  **OR**  **title, abstract, keyword**  **(**(Osteoarthritis OR "Osteoarthritis" OR "Osteoarthritides" OR "Osteoarthrosis" OR "Osteoarthroses" OR "Degenerative Arthritides" OR "Degenerative Arthritis" OR OA) AND (Knee Arthroplasty OR Knee Prosthesis OR "knee replacement arthroplasty" OR "total knee arthroplasty" OR "total knee" OR tka OR "tkr" OR "total knee replacement" OR "knee prosthesis" OR "knee implantation" OR "knee implant" OR "knee implants" OR "knee prosthesis" OR "knee joint replacement" OR "knee joint arthroplasty" OR "Knee Replacement Arthroplasties" OR "Total Knee Replacements" OR "Knee Prostheses" OR "Knee endoprosthesis" OR "Knee endoprostheses" OR "Knee joint arthroplasty" OR "Knee joint arthroplasties" OR "knee joint prosthesis" OR "knee joint prostheses" OR "knee prosthetic" OR "Knee endoprosthetic" OR "knee joint prosthetic" OR "Knee joint endoprosthetic" OR "knee prosthetics" OR "Knee endoprosthetics" OR "knee joint prosthetics" OR "Knee joint endoprosthetics" OR "Knee replacement" OR "Knee replacements" OR "knee arthroplasty" OR "knee arthroplasties" **OR** Hip Arthroplasty OR Hip Prosthesis OR "hip replacement arthroplasty" OR "total hip arthroplasty" OR "total hip" OR tha OR "thr" OR "total hip replacement" OR "hip prosthesis" OR "hip implantation" OR "hip implant" OR "hip implants" OR "hip prosthesis" OR "hip joint replacement" OR "hip joint arthroplasty" OR "Hip Replacement Arthroplasties" OR "Total Hip Replacements" OR "Hip Prostheses" OR "Hip endoprosthesis" OR "Hip endoprostheses" OR "Hip joint arthroplasty" OR "Hip joint arthroplasties" OR "hip joint prosthesis" OR "hip joint prostheses" OR "hip prosthetic" OR "Hip endoprosthetic" OR "hip joint prosthetic" OR "Hip joint endoprosthetic" OR "hip prosthetics" OR "Hip endoprosthetics" OR "hip joint prosthetics" OR "Hip joint endoprosthetics" OR "Hip replacement" OR "Hip replacements" OR "hip arthroplasty" OR "hip arthroplasties") AND ("decision to operate" OR "decision to treat" OR "treatment decision" OR "operation decision" OR "surgery decision" OR "intervention decision" OR "treatment decisions" OR "operation decisions" OR "surgery decisions" OR "intervention decisions" OR Patient Selection OR "patient selection" OR "Patient Selections" OR "Selection for Treatment" OR "Selection for Treatments" OR "Selection of Subjects" OR "Subjects Selection" OR "Subjects Selections" OR "Selection Criteria" OR "priority tool" OR "priority tools" OR "priority criteria" OR "priority criterium" OR "indication set" OR "priority" OR "priorities" OR priorit* OR "indication" OR "indications" OR treatment indication OR "**appropriateness criteria" OR ("evidence" AND ("indication" OR "indications")) OR ((Checklist OR "checklist" OR "checklists") AND ("indication" OR "indications")) OR "guideline" OR "guidelines"))** | 123 |
|  |  |  |
| CENTRAL | title  (Knee Arthroplasty OR Knee Prosthesis OR "knee replacement arthroplasty" OR "total knee arthroplasty" OR "total knee" OR tka OR "tkr" OR "total knee replacement" OR "knee prosthesis" OR "knee implantation" OR "knee implant" OR "knee implants" OR "knee prosthesis" OR "knee joint replacement" OR "knee joint arthroplasty" OR "Knee Replacement Arthroplasties" OR "Total Knee Replacements" OR "Knee Prostheses" OR "Knee endoprosthesis" OR "Knee endoprostheses" OR "Knee joint arthroplasty" OR "Knee joint arthroplasties" OR "knee joint prosthesis" OR "knee joint prostheses" OR "knee prosthetic" OR "Knee endoprosthetic" OR "knee joint prosthetic" OR "Knee joint endoprosthetic" OR "knee prosthetics" OR "Knee endoprosthetics" OR "knee joint prosthetics" OR "Knee joint endoprosthetics" OR "Knee replacement" OR "Knee replacements" OR "knee arthroplasty" OR "knee arthroplasties" **OR** Hip Arthroplasty OR Hip Prosthesis OR "hip replacement arthroplasty" OR "total hip arthroplasty" OR "total hip" OR tha OR "thr" OR "total hip replacement" OR "hip prosthesis" OR "hip implantation" OR "hip implant" OR "hip implants" OR "hip prosthesis" OR "hip joint replacement" OR "hip joint arthroplasty" OR "Hip Replacement Arthroplasties" OR "Total Hip Replacements" OR "Hip Prostheses" OR "Hip endoprosthesis" OR "Hip endoprostheses" OR "Hip joint arthroplasty" OR "Hip joint arthroplasties" OR "hip joint prosthesis" OR "hip joint prostheses" OR "hip prosthetic" OR "Hip endoprosthetic" OR "hip joint prosthetic" OR "Hip joint endoprosthetic" OR "hip prosthetics" OR "Hip endoprosthetics" OR "hip joint prosthetics" OR "Hip joint endoprosthetics" OR "Hip replacement" OR "Hip replacements" OR "hip arthroplasty" OR "hip arthroplasties") AND ("decision to operate" OR "decision to treat" OR "treatment decision" OR "operation decision" OR "surgery decision" OR "intervention decision" OR "treatment decisions" OR "operation decisions" OR "surgery decisions" OR "intervention decisions" OR Patient Selection OR "patient selection" OR "Patient Selections" OR "Selection for Treatment" OR "Selection for Treatments" OR "Selection of Subjects" OR "Subjects Selection" OR "Subjects Selections" OR "Selection Criteria" OR "priority tool" OR "priority tools" OR "priority criteria" OR "priority criterium" OR "indication set" OR "priority" OR "priorities" OR priorit* OR "indication" OR "indications" OR treatment indication OR "**appropriateness criteria" OR ("evidence" AND ("indication" OR "indications")) OR ((Checklist OR "checklist" OR "checklists") AND ("indication" OR "indications")) OR "guideline" OR "guidelines")**  **OR**  **title, abstract, keyword**  **(**(Osteoarthritis OR "Osteoarthritis" OR "Osteoarthritides" OR "Osteoarthrosis" OR "Osteoarthroses" OR "Degenerative Arthritides" OR "Degenerative Arthritis" OR OA) AND (Knee Arthroplasty OR Knee Prosthesis OR "knee replacement arthroplasty" OR "total knee arthroplasty" OR "total knee" OR tka OR "tkr" OR "total knee replacement" OR "knee prosthesis" OR "knee implantation" OR "knee implant" OR "knee implants" OR "knee prosthesis" OR "knee joint replacement" OR "knee joint arthroplasty" OR "Knee Replacement Arthroplasties" OR "Total Knee Replacements" OR "Knee Prostheses" OR "Knee endoprosthesis" OR "Knee endoprostheses" OR "Knee joint arthroplasty" OR "Knee joint arthroplasties" OR "knee joint prosthesis" OR "knee joint prostheses" OR "knee prosthetic" OR "Knee endoprosthetic" OR "knee joint prosthetic" OR "Knee joint endoprosthetic" OR "knee prosthetics" OR "Knee endoprosthetics" OR "knee joint prosthetics" OR "Knee joint endoprosthetics" OR "Knee replacement" OR "Knee replacements" OR "knee arthroplasty" OR "knee arthroplasties" **OR** Hip Arthroplasty OR Hip Prosthesis OR "hip replacement arthroplasty" OR "total hip arthroplasty" OR "total hip" OR tha OR "thr" OR "total hip replacement" OR "hip prosthesis" OR "hip implantation" OR "hip implant" OR "hip implants" OR "hip prosthesis" OR "hip joint replacement" OR "hip joint arthroplasty" OR "Hip Replacement Arthroplasties" OR "Total Hip Replacements" OR "Hip Prostheses" OR "Hip endoprosthesis" OR "Hip endoprostheses" OR "Hip joint arthroplasty" OR "Hip joint arthroplasties" OR "hip joint prosthesis" OR "hip joint prostheses" OR "hip prosthetic" OR "Hip endoprosthetic" OR "hip joint prosthetic" OR "Hip joint endoprosthetic" OR "hip prosthetics" OR "Hip endoprosthetics" OR "hip joint prosthetics" OR "Hip joint endoprosthetics" OR "Hip replacement" OR "Hip replacements" OR "hip arthroplasty" OR "hip arthroplasties") AND ("decision to operate" OR "decision to treat" OR "treatment decision" OR "operation decision" OR "surgery decision" OR "intervention decision" OR "treatment decisions" OR "operation decisions" OR "surgery decisions" OR "intervention decisions" OR Patient Selection OR "patient selection" OR "Patient Selections" OR "Selection for Treatment" OR "Selection for Treatments" OR "Selection of Subjects" OR "Subjects Selection" OR "Subjects Selections" OR "Selection Criteria" OR "priority tool" OR "priority tools" OR "priority criteria" OR "priority criterium" OR "indication set" OR "priority" OR "priorities" OR priorit* OR "indication" OR "indications" OR treatment indication OR "**appropriateness criteria" OR ("evidence" AND ("indication" OR "indications")) OR ((Checklist OR "checklist" OR "checklists") AND ("indication" OR "indications")) OR "guideline" OR "guidelines"))** | 7 |
|  |  |  |
| CINAHL | title  (Knee Arthroplasty OR Knee Prosthesis OR "knee replacement arthroplasty" OR "total knee arthroplasty" OR "total knee" OR tka OR "tkr" OR "total knee replacement" OR "knee prosthesis" OR "knee implantation" OR "knee implant" OR "knee implants" OR "knee prosthesis" OR "knee joint replacement" OR "knee joint arthroplasty" OR "Knee Replacement Arthroplasties" OR "Total Knee Replacements" OR "Knee Prostheses" OR "Knee endoprosthesis" OR "Knee endoprostheses" OR "Knee joint arthroplasty" OR "Knee joint arthroplasties" OR "knee joint prosthesis" OR "knee joint prostheses" OR "knee prosthetic" OR "Knee endoprosthetic" OR "knee joint prosthetic" OR "Knee joint endoprosthetic" OR "knee prosthetics" OR "Knee endoprosthetics" OR "knee joint prosthetics" OR "Knee joint endoprosthetics" OR "Knee replacement" OR "Knee replacements" OR "knee arthroplasty" OR "knee arthroplasties" **OR** Hip Arthroplasty OR Hip Prosthesis OR "hip replacement arthroplasty" OR "total hip arthroplasty" OR "total hip" OR tha OR "thr" OR "total hip replacement" OR "hip prosthesis" OR "hip implantation" OR "hip implant" OR "hip implants" OR "hip prosthesis" OR "hip joint replacement" OR "hip joint arthroplasty" OR "Hip Replacement Arthroplasties" OR "Total Hip Replacements" OR "Hip Prostheses" OR "Hip endoprosthesis" OR "Hip endoprostheses" OR "Hip joint arthroplasty" OR "Hip joint arthroplasties" OR "hip joint prosthesis" OR "hip joint prostheses" OR "hip prosthetic" OR "Hip endoprosthetic" OR "hip joint prosthetic" OR "Hip joint endoprosthetic" OR "hip prosthetics" OR "Hip endoprosthetics" OR "hip joint prosthetics" OR "Hip joint endoprosthetics" OR "Hip replacement" OR "Hip replacements" OR "hip arthroplasty" OR "hip arthroplasties") AND ("decision to operate" OR "decision to treat" OR "treatment decision" OR "operation decision" OR "surgery decision" OR "intervention decision" OR "treatment decisions" OR "operation decisions" OR "surgery decisions" OR "intervention decisions" OR Patient Selection OR "patient selection" OR "Patient Selections" OR "Selection for Treatment" OR "Selection for Treatments" OR "Selection of Subjects" OR "Subjects Selection" OR "Subjects Selections" OR "Selection Criteria" OR "priority tool" OR "priority tools" OR "priority criteria" OR "priority criterium" OR "indication set" OR "priority" OR "priorities" OR priorit* OR "indication" OR "indications" OR treatment indication OR "**appropriateness criteria" OR ("evidence" AND ("indication" OR "indications")) OR ((Checklist OR "checklist" OR "checklists") AND ("indication" OR "indications")) OR "guideline" OR "guidelines")**  **OR**  **title, abstract, keyword**  **(**(Osteoarthritis OR "Osteoarthritis" OR "Osteoarthritides" OR "Osteoarthrosis" OR "Osteoarthroses" OR "Degenerative Arthritides" OR "Degenerative Arthritis" OR OA) AND (Knee Arthroplasty OR Knee Prosthesis OR "knee replacement arthroplasty" OR "total knee arthroplasty" OR "total knee" OR tka OR "tkr" OR "total knee replacement" OR "knee prosthesis" OR "knee implantation" OR "knee implant" OR "knee implants" OR "knee prosthesis" OR "knee joint replacement" OR "knee joint arthroplasty" OR "Knee Replacement Arthroplasties" OR "Total Knee Replacements" OR "Knee Prostheses" OR "Knee endoprosthesis" OR "Knee endoprostheses" OR "Knee joint arthroplasty" OR "Knee joint arthroplasties" OR "knee joint prosthesis" OR "knee joint prostheses" OR "knee prosthetic" OR "Knee endoprosthetic" OR "knee joint prosthetic" OR "Knee joint endoprosthetic" OR "knee prosthetics" OR "Knee endoprosthetics" OR "knee joint prosthetics" OR "Knee joint endoprosthetics" OR "Knee replacement" OR "Knee replacements" OR "knee arthroplasty" OR "knee arthroplasties" **OR** Hip Arthroplasty OR Hip Prosthesis OR "hip replacement arthroplasty" OR "total hip arthroplasty" OR "total hip" OR tha OR "thr" OR "total hip replacement" OR "hip prosthesis" OR "hip implantation" OR "hip implant" OR "hip implants" OR "hip prosthesis" OR "hip joint replacement" OR "hip joint arthroplasty" OR "Hip Replacement Arthroplasties" OR "Total Hip Replacements" OR "Hip Prostheses" OR "Hip endoprosthesis" OR "Hip endoprostheses" OR "Hip joint arthroplasty" OR "Hip joint arthroplasties" OR "hip joint prosthesis" OR "hip joint prostheses" OR "hip prosthetic" OR "Hip endoprosthetic" OR "hip joint prosthetic" OR "Hip joint endoprosthetic" OR "hip prosthetics" OR "Hip endoprosthetics" OR "hip joint prosthetics" OR "Hip joint endoprosthetics" OR "Hip replacement" OR "Hip replacements" OR "hip arthroplasty" OR "hip arthroplasties") AND ("decision to operate" OR "decision to treat" OR "treatment decision" OR "operation decision" OR "surgery decision" OR "intervention decision" OR "treatment decisions" OR "operation decisions" OR "surgery decisions" OR "intervention decisions" OR Patient Selection OR "patient selection" OR "Patient Selections" OR "Selection for Treatment" OR "Selection for Treatments" OR "Selection of Subjects" OR "Subjects Selection" OR "Subjects Selections" OR "Selection Criteria" OR "priority tool" OR "priority tools" OR "priority criteria" OR "priority criterium" OR "indication set" OR "priority" OR "priorities" OR priorit* OR "indication" OR "indications" OR treatment indication OR "**appropriateness criteria" OR ("evidence" AND ("indication" OR "indications")) OR ((Checklist OR "checklist" OR "checklists") AND ("indication" OR "indications")) OR "guideline" OR "guidelines"))** | 51 |
|  |  |  |
| Total |  | 3065 |
